# Single bout of exercise triggers the increase of vitamin D blood concentration in adolescent trained boys: a pilot study

**DOI:** 10.1038/s41598-022-05783-x

**Published:** 2022-02-03

**Authors:** Katarzyna Patrycja Dzik, Tomasz Grzywacz, Marcin Łuszczyk, Sylwester Kujach, Damian Józef Flis, Jan Jacek Kaczor

**Affiliations:** 1 Department of Physiology and Biochemistry, Gdansk University of Physical Education and Sport, K. Gorskiego 1, 80-336 Gdansk, Poland; 2 Department of Animal and Human Physiology, Faculty of Biology, University of Gdansk, Jana Bażyńskiego 8, 80-309 Gdansk, Poland; 3 Department of Sport, Institute of Physical Education, Kazimierz Wielki University, Chodkiewicza 30, 85-064 Bydgoszcz, Poland; 4 Department of Human Physiology, Faculty of Medicine, Medical University of Gdansk, Tuwima 15, 80-210 Gdansk, Poland; 5 Department of Pharmaceutical Pathophysiology, Faculty of Pharmacy, Medical University of Gdansk, Debinki 7, 80-211 Gdansk, Poland

**Keywords:** Physiology, Endocrinology

## Abstract

Vitamin D is necessary for musculoskeletal health, however, the supplementation of vitamin D above the sufficiency level does not bring additional bone mass density (BMD), unlike physical exercise which enhances the bone formatting process. Regular physical activity has been shown to upregulate VDR expression in muscles and to increase circulating vitamin D. Here we investigate whether a single bout of exercise might change 25(OH)D_3_ blood concentration and how it affects metabolic response to exercise. Twenty-six boys, 13.8 years old (SD ± 0.7) soccer players, participated in the study. The participants performed one of two types of exercise: the first group performed the VO_2_max test until exhaustion, and the second performed three times the repeated 30 s Wingate Anaerobic Test (WAnT). Blood was collected before, 15 min and one hour after the exercise. The concentration of 25(OH)D_3_, parathyroid hormone (PTH), interleukin-6 (IL-6), lactate, non-esterified fatty acids (NEFA) and glycerol were determined. 25(OH)D_3_ concentration significantly increased after the exercise in all boys. The most prominent changes in 25(OH)D_3_, observed after WAnT, were associated with the rise of PTH. The dimensions of response to the exercises observed through the changes in the concentration of 25(OH)D_3_, PTH, NEFA and glycerol were associated with the significant increases of IL-6 level. A single bout of exercise may increase the serum’s 25(OH)D_3_ concentration in young trained boys. The intensive interval exercise brings a more potent stimulus to vitamin D fluctuations in young organisms. Our results support the hypothesis that muscles may both store and release 25(OH)D_3_.

## Introduction

Vitamin D has been the subject of an enormous number of studies in the past decade. Although it is stated that vitamin D is necessary for musculoskeletal health, recent meta-analyses do not show any direct effect of vitamin D supplementation on muscle strength or athlete performance even though that serum 25(OH)D_3_ concentrations are placed after supplementation at the sufficiency level ^1,2^. Therefore, it appears that the role of vitamin D extends to the regulatory factor rather than the anabolic or performance improvement factor. Vitamin D does not seem to have a definable tissue storage site. The animal study has shown that 25(OH)D_3_ was stored 33% in fat and 20% in muscle ^3^. Human studies have shown that adipose tissue is a storage site for vitamin D ^4,5^. However, vitamin D might not be released separately, only when stored fatty acids are mobilized to supply energy ^6,7^. The presence of vitamin D receptor (VDR) ^8^, as well as the activity of 25-hydroxyvitamin D-1-α-hydroxylase (CYP27B1) and cytochrome P450 family 24 subfamily A member 1 (CYP24A1) enzymes ^9,10^ have been confirmed in skeletal muscle studies, which suggests the possible local regulation and synthesis of vitamin D in muscles ^9,11^. Abboud et al. ^12^ conducted experimented with the concentration and time-dependent effects of calcitriol on the capacity of muscle cells to take up and release 25(OH)D_3_, which confirms the presence of 25(OH)D_3_ in skeletal muscle cells. They suggested that muscular 25(OH)D_3_, which is protected from inactivating by CYP24A1 in the liver, has the potential to increase the concentration of circulating 25(OH)D_3_ especially when vitamin D status is falling in winter ^13,14^.

When activated, vitamin D is responsible for increasing calcium absorption from the intestines ^15^, which is a foundation for a proper bone formation process ^16,17^. However, meta-analyses stated that vitamin D supplementation contributes to the improvements in bone mineral density (BMD) in children and adolescents but only in the cases of vitamin D deficiency. In contrast, it is unlikely that vitamin D supplements are beneficial in children and adolescents with normal vitamin D concentrations ^18,19^. On the other hand, unlike vitamin D, regular weight-bearing exercises reveal the ability to improve bone mineral accretion in healthy children ^20^ and achieve an optimum peak bone mass level due to the positive osteogenic response ^21,22^. Nevertheless, the mechanical loading of the exercise is a major regulator of stimulus for the bone formatting process since non-impact loading sports, for instance, swimming, do not display any significant impact on bone mineral density ^23–25^.

Progression through puberty includes rapid and significant changes in many physiological processes, including carbohydrate and lipid metabolism. Whereas the activities of the anaerobic enzymes such as creatine kinase, phosphofructokinase, lactate dehydrogenase are lower in children (3–11 years) as compared to adults ^26^, the activities of the aerobic enzymes (succinate dehydrogenase, fumarase) are higher in children compared to adults ^27^. In addition, relative fat oxidation is higher in children than adults ^28^. However, children aged 13–15 years demonstrate similar phosphofructokinase, lactate dehydrogenase, and citrate synthase activities compared to adults ^29^, which suggests a fine line in the metabolism transformation during puberty. Therefore, lipids and lactate monitoring are necessary to understand the metabolic processes of exercise during puberty.

In 1961, Goldstein hypothesized a “muscle humoral factor” released into the circulation during exercise due to muscle fiber damage ^30^. Nowadays, the human secretory myokinome comprises hundreds of factors ^31,32^ even though just 5% of the myokinome has been ascribed to specific functions ^33^. Although exercise triggers the release of myokines from the skeletal muscle and exerkines from other tissues into circulation ^34^, the myokine signature is emerging to be fiber-type specific, and its expression/regulation might depend on exercise type, protocol, and duration (i.e., training vs. acute exercise) ^31^. In addition, a recent study of human skeletal muscle suggested the possible crosstalk between vitamin D and IL-6 since the increase of IL-6 induced by nutrient restriction appeared to be dependent on vitamin D ^35^.

There are multi-faceted links between vitamin D status and musculoskeletal health. In the present study, we investigate the metabolic response to a single bout of exercise in young boys regarding fat consumption, lactate production, IL-6, and PTH production, and the possible vitamin D fluctuations. We hypothesize that exercise triggers the release of vitamin D stored in skeletal muscles.

## Methods

### Study design

Twenty-six boys, 13.8 years old (SD ± 0.7) soccer players, participated in the study. The study was conducted in accordance with the Declaration of Helsinki, and the experimental protocol was approved by the local institutional Bioethical Committee in Gdansk (No. NKBBN/455/2015). The parents of child participants provided informed written voluntary consent for the investigation. The participants were randomly divided into two groups. The first group (VO_2max_ group) performed the VO_2max_ test until exhaustion, and the second one (WAnT group) performed three times repeated 30 s Wingate Anaerobic Test. The subjects were included in the study if they were considered healthy normal weight, did not suffer from any chronic conditions, and passed a physical examination. Boys had practiced soccer 2–3 times per week for at least 4 years before the experiment. All the participants were instructed to withdraw from all high-intensity workouts at least 48 h before the testing session. The basic anthropometric and physiological characteristics of the subjects are summarized in Table 1.

**Table 1:** The morphological and physiological characteristics of the participants. Values are mean ± SD expressed in absolute or relative values; Fat (%), fat percentage; FFM, free fatty mass; BMI, body mass index; WBC, white blood cells; RBC, red blood cells; LA, lactic acid.

	All (n = 26)	WAnT group			VO_2_ max test group		
		All (n = 14)	Pre-pubertal (n = 7)	Pubertal (n = 7)	All (n = 12)	Pre-pubertal (n = 5)	Pubertal (n = 7)
Age [years]	13.8 ± 0.7	13.9 ± 0.4	13.5 ± 1.3	13.9 ± 0.3	13.7 ± 1.0	14.0 ± 0.5	13.8 ± 0.3
Biological age [years]	14.2 ± 1.9	14.5 ± 1.9	12.6 ± 1.4	15.1 ± 1.4	13.9 ± 1.9	13.1 ± 0.9	15.5 ± 1.8
Weight [kg]	52.8 ± 10.0	54.3 ± 10.9	44.8 ± 6.7	56.7 ± 7.1	50.7 ± 9.1	46.8 ± 4.9	59.6 ± 11.2
Height [cm]	167.6 ± 10.7	169.0 ± 10.6	158.6 ± 9.8	173.0 ± 6.3	165.8 ± 10.9	161.6 ± 6.6	174.29 ± 9.9
FAT %	9.3 ± 2.6	9.5 ± 2.8	8.7 ± 2.2	8.9 ± 2.82	8.8 ± 2.4	8.7 ± 2.5	10.07 ± 3.11
FFM [kg]	47.8 ± 8.3	48.9 ± 9.1	40.9 ± 6.1	51.4 ± 4.9	46.2 ± 7.7	42.7 ± 3.9	53.4 ± 9.2
BMI [kg m^−2^]	18.6 ± 1.5	18.8 ± 1.7	17.7 ± 1.1	18.9 ± 1.5	18.3 ± 1.4	17.9 ± 1.2	19.5 ± 1.7
WBC [G L^−1^]	5.7 ± 1.4	5.9 ± 1.5	5.6 ± 1.5	6.3 ± 1.6	5.3 ± 1.3	5.8 ± 1.4	5.0 ± 1.2
RBC [T L^−1^]	4.8 ± 0.3	4.8 ± 0.3	4.7 ± 0.3	4.9 ± .03	4.8 ± 0.3	4.7 ± 0.3	4.9 ± 0.3
Hemoglobin [g dL^−1^]	13.6 ± 0.9	13.5 ± 0.9	13.4 ± 1.1	13.7 ± 0.8	13.7 ± 0.9	13.6 ± 0.9	13.8 ± 0.9
LA before test [mmol L^−1^]	2.1 ± 0.8	2.3 ± 0.9	2.3 ± 0.6	2.4 ± 1.2	1.8 ± 0.5	2.0 ± 0.6	1.6 ± 0.2
LA after 1. round [mmol L^−1^]		9.9 ± 1.3	9.76 ± 0.8	10.0 ± 1.8			
LA after 2. round [mmol L^−1^]		10.5 ± 1.2	10.4 ± 1.0	10.5 ± 1.4			
LA 3 min after test [mmol L^−1^]	5.9 ± 2.3	7.6 ± 1.2	8.0 ± 1.2	7.2 ± 1.1	3.4 ± 0.9	3.3 ± 0.8	3.6 ± 1.1
LA 15 min after test [mmol L^−1^]	5.8 ± 3.7	8.6 ± 2.5	8.5 ± 2.8	8.7 ± 2.2	1.9 ± 0.4	1.73 ± 0.3	2.0 ± 0.06
LA 1 h after test [mmol L^−1^]	2.6 ± 1.1	3.2 ± 1.3	3.0 ± 1.4	3.4 ± 1.3	1.6 ± 0.1	1.55 ± 0.15	1.6 ± .06

The boys’ biological age was calculated according to appropriate weight and height percentile grids developed by the Mother and Child Institute, Warsaw, Poland ^36^. Boys whose biological age was higher than their chronological age were included in the pubertal group, and boys with biological age equal to or lower than their chronological age were included in the pre-pubertal group. The allocation to the pubertal and pre-pubertal groups was done after the randomization to VO_2max_ and WAnT groups.

### Body composition assessment

The amount of body water and body composition, including body mass, fat mass, fat free mass (FFM), skeletal muscle mass, and soft lean mass, was measured using a multi-frequency impedance plethysmograph body composition analyzer (InBody 720, Biospace Analyzer, Korea).

### Maximal Oxygen Uptake (VO_2max_) Test

The VO_2max_ group performed a graded test on an electromagnetically braked cycle ergometer (ER 900 Jaeger, Germany/Viasys Health Care). The participants were allowed a 5-min warm-up period at an intensity of 1.5 W kg^−1^, with a pedaling cadence of 60 rpm. Immediately following the warm-up, the participants began VO_2max_ testing by cycling at increasingly workloads by 25 W min^−1^ until the participant reached the point of volitional exhaustion ^37^. The recovery was passive in a seated position. VO_2max_ was determined when at least two of the following criteria were satisfied: (1) the respiratory exchange ratio (RER) exceeded 1.05, (2) achievement of 90% of age-predicted peak HR (220–age), and (3) ratings of perceived exertion (RPE) of 19 or 20 ^38^. Breath-by-breath pulmonary gas exchange was measured (Oxycon-Pro, Jaeger-Viasys Health Care, Hochberg, Germany) throughout the VO_2max_ test; the O_2_ and CO_2_ analyzers were calibrated prior to each test using standard gases of known concentrations by manufacturer guidelines. All participants performed the tests at a similar time in the morning, at least 3 h after light breakfast.

### Wingate Anaerobic Test (WAnT)

The WAnT group performed the Wingate Anaerobic Test on a mechanically braked cycle ergometer (884E Sprint Bike, Monark, Sweden). Anaerobic capacity protocol started with a standard 5 min warm-up at 1.0 (W kg^−1^) of body mass (BM). After the warm-up subject performed a 30 s all-out supra-maximal task, followed by 5 min rest and repeated overall three times. Flywheel resistance equaled 0.075 kG per kg of BM (corresponding to 7.5% of the participant’s BM). The participat initiated the test from a dead stop. The resistance on the ergometer’s friction belt had been preset by the testers immediately before WAnT. For the analysis, PP—peak power (W kg^−1^) and total work—W tot (J kg^−1^) were taken into account. All the tests described above were performed in similar times in the morning, at least 3 h after a light breakfast.

### Blood collection and analysis

Blood samples were taken three times from the antecubital vein into the vacutainer tubes with a silica clot activator before, 15 min, and one hour after the tests. Additionally, in the WAnT group, the blood samples were taken 3 min after the first and second bout for lactate measurement. The samples were centrifuged at 2000*g* for 10 min at 4 °C. The separated serum and plasma samples were frozen and kept at − 80 °C until later analysis. The tubes containing the samples were number-coded to blind the laboratory personnel regarding the treatment group and the sequence of sample collection.

Serum 25(OH)D_3_ concentration was determined by enzyme immunoassay method using 25-OH Vitamin D total ELISA kit (DE1971, Demeditec Diagnostics, Germany), according to the manufacturer’s instructions.

Plasma interleukin 6 (IL-6) concentration was determined by ELISA using a commercial kit (HS600, R&D Systems, USA).

Plasma parathyroid hormone (PTH) was determined using PTH intact ELISA kit (DE 3645, Demeditec Diagnostics, Germany).

Plasma non-esterified fatty acids and glycerol concentrations were determined by direct colorimetric methods using NEFA assay (FA115, Randox, United Kingdom) and Glycerol assay (GY105, Randox, United Kingdom).

### Statistical calculations

Statistical analyses were performed using STATISTICA 13.0. Results are expressed as the mean, standard deviation (x ± SD) for demographic and clinical characteristics, and mean and standard error (x ± SEM) the rest for the parameters. The normality of data was tested using the Shapiro–Wilks W-test. The level of significance was set at 0.05 for all analyses. The differences between obtained data before, 15 min, and one hour after the exercises were analyzed using repeated measures of variance (ANOVA) for parametric variables and the U-Mann Whitney test for nonparametric ones. Associations among measured parameters were analyzed using Pearson’s linear regression (coefficient, r). Post hoc testing for specific differences was performed using the Least Significant Difference test. All data are given as means ± SEM, *p* < 0.05 was considered significant.

### Ethics approval

The study was conducted in accordance with the Declaration of Helsinki, and the experimental protocol was approved by the local institutional Bioethical Committee in Gdansk (No. NKBBN/455/2015).

### Consent to participate

Written informed consent was obtained from participants’ parents.

## Results

**Figure 1:**
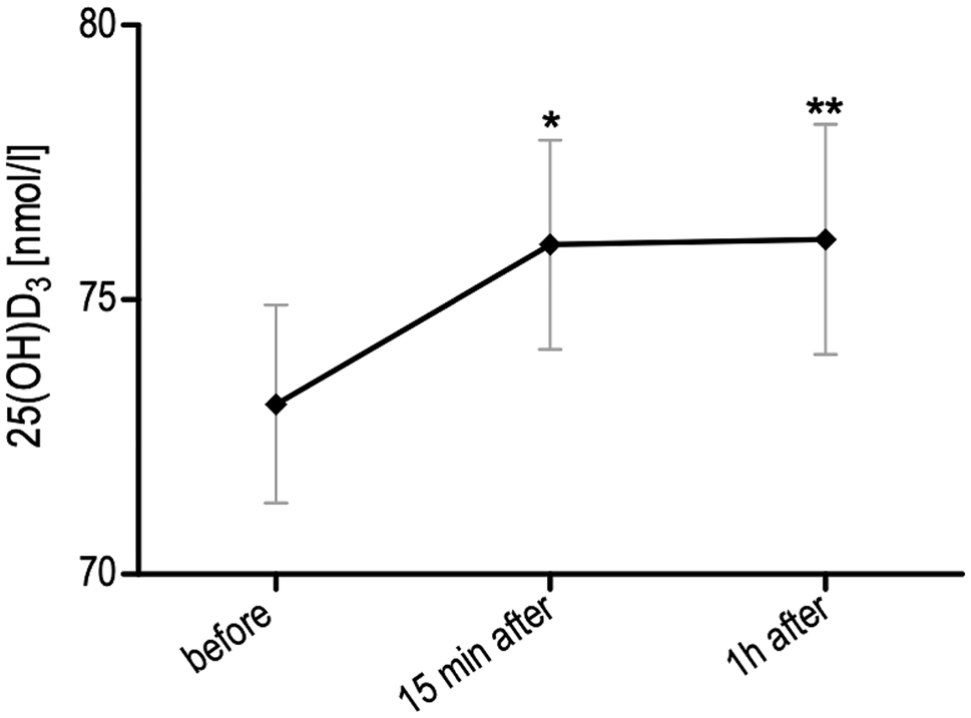
The concentration of 25(OH)D_3_ before, 15 min after and 1 h after a single bout of exercise (both VO_2max_ and WAnT tests) in boys n = 26 (10–14 years). Results were expressed as mean ± SEM. *p = 0.016—difference between indicated result/mean and the results before, **p = 0.011—difference between indicated result/mean the results before, LSD post hoc test after repeated measures ANOVA.

All boys had a baseline vitamin D concentration above 50 nmol L^−1^. The serum concentration of 25(OH)D_3_ increased average by 2.9 and 3.0 nmol L^−1^, respectively 15 min (p = 0.016) and 1 h (p = 0.011) after the exercise in all boys (Fig. 1).

**Figure 2:**
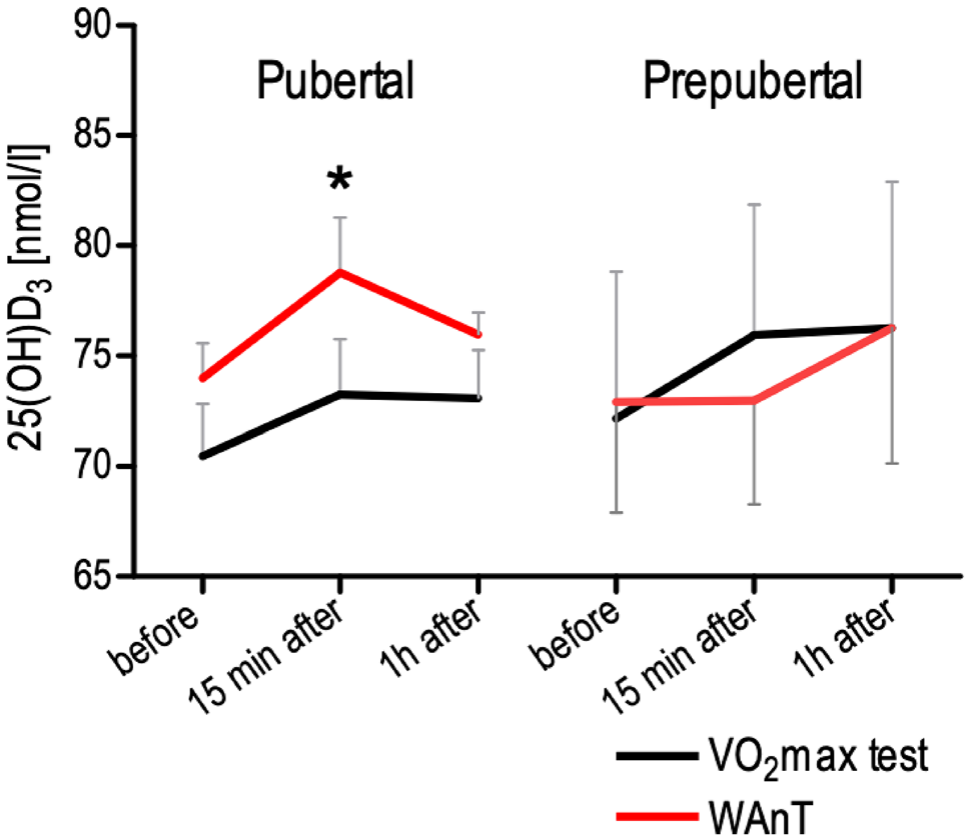
The concentration of 25(OH)D3 before, 15 min after and 1 h after a single bout of exercise in pubertal (n = 7 for VO_2max_ test, and n = 7 for WAnT test) and prepubertal (n = 5 for VO_2max_ test, and n = 7 for WAnT test) boys according to two types of exercise (VO_2_max and WAnT tests). *p = 0.032—difference between indicated result/mean and the results before in the same group and test, LSD post hoc test after repeated measures ANOVA.

Considering different types of exercise, we did not observe a significant difference between the tests in all boys. However, taking into account the biological age of the boys it was found a significant rise 15 min after WAnT (p = 0.032) in pubertal boys; it was 73.98 ± 1.59, 78.78 ± 2.51 15 min after, and 75.96 ± 1.00 nmol L^−1^ 1 h after the exercise. The concentration of 25(OH)D_3_ increased in both pre-pubertal and pubertal boys 15 min after the VO_2max_ test and dropped one hour after the exercise, but it was not significantly different at the particular time points. The only group in which 25(OH)D_3_ concentration increased one hour after instead of 15 min after the exercise was the pre-pubertal WAnT group. 25(OH)D_3_ concentration in this group was 72.90 ± 4.92, 72.86 ± 4.67 15 min after and 76.29 ± 6.16 nmol L^−1^ 1 h after the test (Fig. 2).

**Figure 3:**
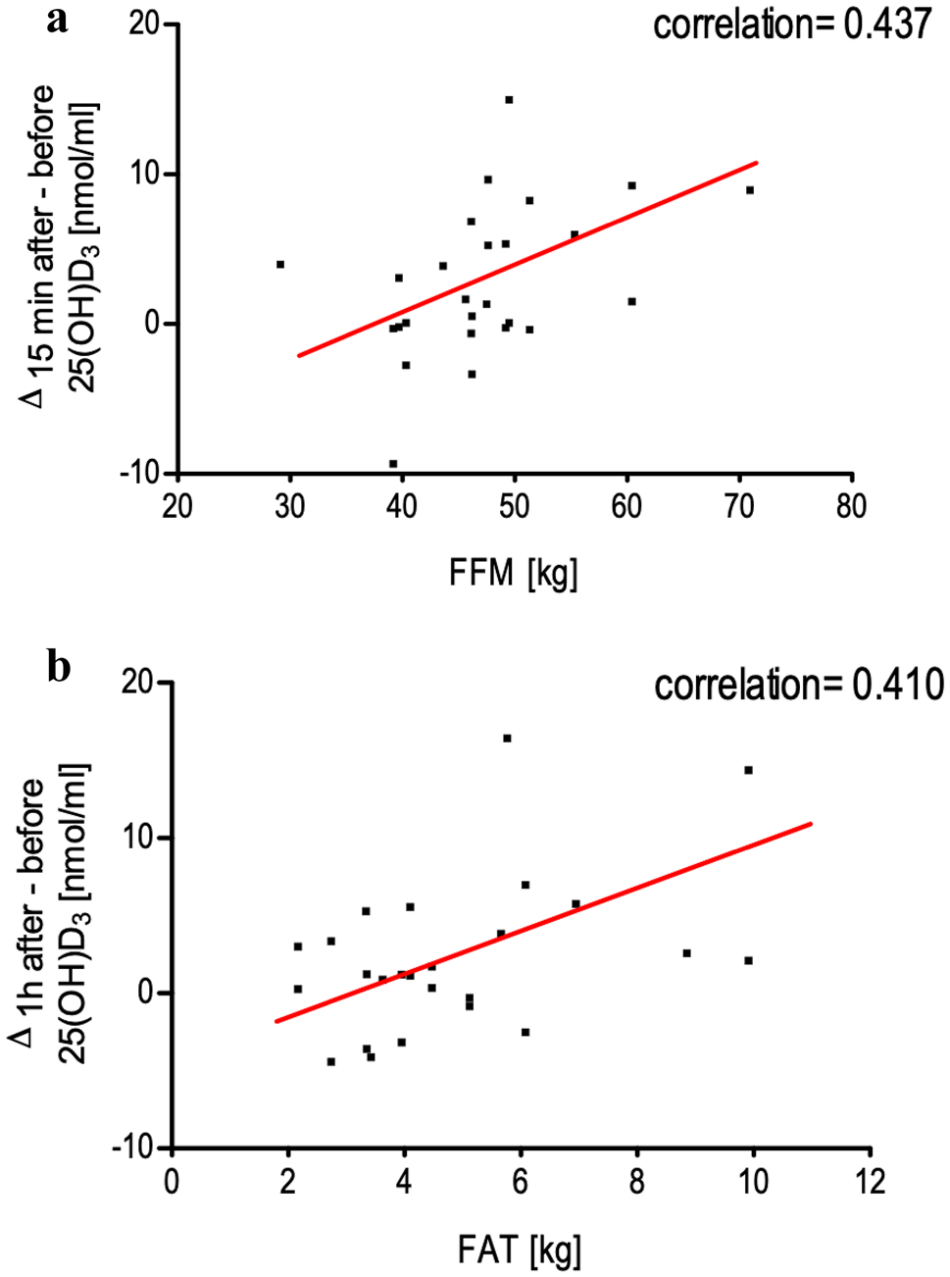
(**a**) The correlation between the delta change in 25(OH)D_3_ before and 15 min after the exercise and free fatty mass (FFM) in all boys. (**b**) The correlation between the delta change in 25(OH)D_3_ before and 1 h after a single bout of exercise and fat [kg] in all boys. For (**a**) and (**b**) n = 26 (10–14 years), Pearson’s linear regression.

Delta of changes in vitamin D concentration before and 15 min after the exercise positively correlated to FFM (0.44) (p = 0.026) (Fig. 3a). We did not observe any association between the delta of changes in vitamin D before and 15 min after the exercise with fat storage (FAT % or FAT [kg]). However, the delta of changes in vitamin D concentration before and 1 h after the exercise positively correlates with the amount of fat storage, both FAT % and FAT [kg], but had no connection to FFM [kg] (Fig. 3b).

**Figure 4:**
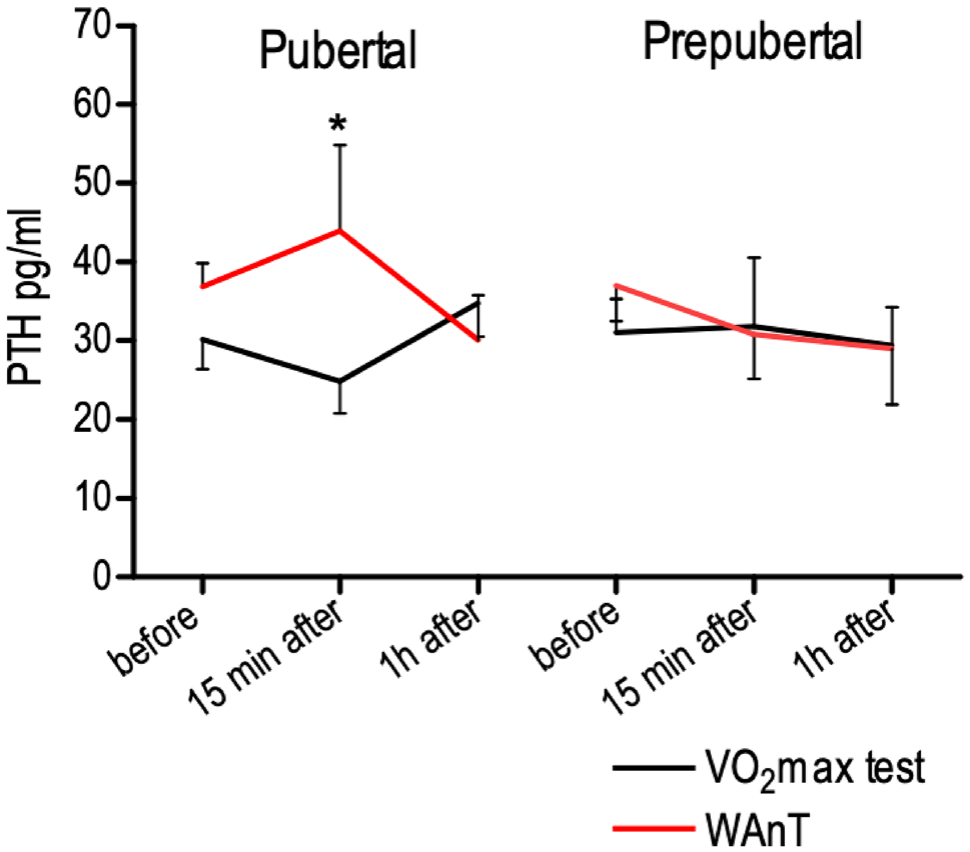
The PTH concentration before, 15 min after and 1 h after a single bout of exercise in pubertal (n = 7 for VO_2_max test, and n = 7 for WAnT test) and prepubertal (n = 5 for VO_2_max test, and n = 7 for WAnT test) boys according to two types of exercise (VO_2_max and WAnT tests). *p = 0.024—difference between indicated result/mean and the results 1 h after in the same group and test, LSD post hoc test after repeated measures ANOVA.

PTH concentration increased 15 min after exercise in the pubertal group performing WAnT from 36.84 ± 2.97 to 43.91 ± 10.92 and then significantly (p = 0.030) dropped to 30.04 ± 5.66 pg mL^−1^ 1 h after the test. Interestingly, 15 min after the VO_2_ max test, the PTH concentration decreased from 30.12 ± 3.81 to 24.85 ± 4.10 pg mL^−1^ in the pubertal group and is significantly (p = 0.024) lower from PTH concentration 15 min after the WAnT. In pre-pubertal group, we did not find any significant changes at the different time points (Fig. 4).

IL-6 concentration significantly increased 15 min after both tests in all boys (p = 0.003), yet this tendency was continued until one hour after the exercise only in the WAnT group (p = 0.015), while one hour after VO_2_ max test, IL-6 concentration dropped to the baseline. IL-6 concentration in the WAnT group was 0.56 ± 0.08 before, 0.89 ± 0.11 15 min after, and 1.25 ± 0.25 pg mL^−1^ 1 h following the test. In the VO_2_ max test group IL-6 concentration was 0.61 ± 0.18 before, 0.95 ± 0.24 15 min after, and 0.78 ± 0.15 pg mL^−1^ 1 h following the test (Table 2).

**Table 2:** Il-6, NEFA and glycerol level at baseline, 15 min and 1 h after the tests.

	WAnT group (n = 14)			VO_2_ max test group (n = 12)		
	All	Pre-pubertal (n = 7)	Pubertal (n = 7)	All	Pre-pubertal (n = 5)	Pubertal (n = 7)
**Il-6 [pg ml**^**−1**^**]**						
Before	0.56 ± 0.08^*3*^	0.54 ± 0.11^*3*^	0.57 ± 0.12^*3*^	0.61 ± 0.18	0.36 ± 0.10	0.79 ± 0.29
15 min after	0.89 ± 0.11^*1*^	0.88 ± 0.12^*1*^	0.90 ± 0.19	0.95 ± 0.24^*1*^	0.67 ± 0.06^*1*^	1.15 ± 0.40
1 h after	1.25 ± 0.25^*2*^	0.97 ± 0.17	1.52 ± 0.45^*2*^	0.78 ± 0.15	0.58 ± 0.07^*4*^	0.93 ± 0.24
**NEFA [mmol mL**^**−1**^**]**						
Before	0.093 ± 0.02	0.110 ± 0.03	0.076 ± 0.02	0.155 ± 0.05	0.236 ± 0.10	0.097 ± 0.02
15 min after	0.084 ± 0.02	0.085 ± 0.02	0.083 ± 0.03	0.160 ± 0.05	0.251 ± 0.10	0.095 ± 0.02
1 h after	0.192 ± 0.07^*2*^	0.109 ± 0.02	0.276 ± 0.14^*2,3*^	0.193 ± 0.05	0.274 ± 0.11	0.136 ± 0.04
**Glycerol [μmol mL**^**−1**^**]**						
Before	11.13 ± 1.18	12.81 ± 2.03	9.44 ± 0.97	12.93 ± 1.85	15.51 ± 4.13	11.09 ± 1.14
15 min after	54.91 ± 4.72^*1,2*^	64.96 ± 4.42^*1,2,5*^	44.86 ± 6.58^*1,2,5*^	18.70 ± 3.41^*4*^	23.96 ± 6.36^*4*^	14.95 ± 3.40^*4*^
1 h after	27.71 ± 4.42^*3*^	28.59 ± 6.93^*3*^	26.82 ± 6.03^*3*^	12.86 ± 1.72^*4*^	15.81 ± 3.37	10.76 ± 1.45^*4*^

^1^p < 0.05 between results before and 15 min after in the same group.

^2^p < 0.05 between 15 min after to 1 h after in the same group.

^3^p < 0.05 between before and 1 h after in the same group.

^4^p < 0.05 between WAnT at the same time point in the same group.

^5^p < 0.05 between pubertal and pre-pubertal groups at the same time points in the same tests.

IL-6 concentration changes were similar in both pubertal and pre-pubertal groups, although slightly more striking in the older group. The significant difference regarding IL-6 concentration between pre-pubertal and pubertal boys in the WAnT group was detected one hour the exercise wherein the pubertal group IL-6 concentration reached 1.52 ± 0.45 while in pre-pubertal boys it was 0.97 ± 0.17 pg mL^−1^. Similarly, in the VO_2_ max test group, pubertal boys had higher IL-6 concentration as compared to the pre-pubertal group 15 min after the test; in the pubertal group, IL-6 concentration was 1.15 ± 0.40 and 0.67 ± 0.06 pg mL^−1^ in the pre-pubertal group (Table 2).

Plasma NEFA concentration changed after both tests; yet again the more distinguished differences were observed in the WAnT group in pubertal boys. Fifteen minutes after the exercise in pubertal boys, we observed a slightly elevated NEFA concentration, while in pre-pubertal boys, NEFA concentration decreased. Interestingly, in older boys, one hour after WAnT, NEFA concentration increased by 3.6 times as compared to the baseline (p = 0.002), while at the same time, in pre-pubertal boys, it was already at the baseline level. In the VO_2max_ test group, the highest NEFA release was found one hour after the test in both pubertal and prepubertal boys, but the increase was not higher than 42% of the baseline NEFA concentration (Table 2).

Glycerol concentration drastically shifted 15 min after WAnT in both pubertal and pre-pubertal boys. At the baseline, it was 11.13 ± 1.18 μmol mL^−1^, 15 min after the exercise it increased by ∼ 5.0 times and reached 54.91 ± 4.72 μmol mL^−1^ (p = 0.00001), and one hour after the exercise it dropped to the 27.71 ± 4.42 μmol mL^−1^ (p = 0.00008). Surprisingly, in the pre-pubertal group, the glycerol concentration 15 min after the WAnT was significantly higher than in the pubertal boys at the same time point (p = 0.001) (Table 2).

After the VO_2max_ test, glycerol concentration slightly increased 15 min after the exercise, but the increase was significantly lower than after the WAnT in pre-pubertal (p = 0.002) and pubertal boys (p = 0.0000006). One hour after the VO_2_ max test, glycerol concentration was already at the baseline level in all boys (Table 2).

## Discussion

The main finding of our study is the increase in the 25(OH)D_3_ concentration after the exercise in all of the groups. However, the changes over time and extension of the rises differ among pre-pubertal and pubertal boys and regarding to the type of exercise. Additionally, the most distinguished increase in 25(OH)D_3_ concentration in pubertal boys after the WAnT was observed along with a significant increase in PTH concentration. Moreover, we noticed a clear contribution of lipid metabolism in both WAnT and VO_2max_ test recognizable through the alteration in the glycerol and NEFA, yet the prominent post-exercise lipolysis intensification was observed mainly after the WAnT. At last, the dimensions of response to the exercises observed through the changes in the level of 25(OH)D_3_, PTH, NEFA, and glycerol were associated with the significant increases of IL-6 concentration in both pubertal and pre-pubertal boys. The above changes suggest that intensive interval exercise brings a more potent stimulus to vitamin D secretion in young organisms.

Our hypothesis states that the exercise might act as the trigger to release the 25(OH)D_3_ to the circulation. In present study, we show the increase of 25(OH)D_3_ concentration by the average of 2.9 and 3.0 nmol L^−1^, 15 min and one hour after the exercise, respectively. Previously, a study on postmenopausal women showed that 12-week of moderate-intensity aerobic exercise, with no additional vitamin D supplementation, increased serum vitamin D concentration, as severe vitamin D deficiency status (below 10 ng mL^−1^) was improved to vitamin D deficiency status (between 10 and 20 ng mL^−1^) ^39^. The recent investigation also reported that 20 weeks of human resistant training, without vitamin D supplementation, increased plasma 25(OH)D_3_ concentration from 42.4 to 51.2 nmol L^−1^, as well it induced the expression of CYP27B1, which positively correlated with an up-regulation of VDR ^9^. Those findings suggest, that the increase of the 25(OH)D_3_ after a single bout of exercise might be maintained and enhanced by the training process.

Based on our previously published results ^40^ and present investigation, we suggest that VDR may potentially regulate local control of vitamin D metabolism in the skeletal muscle. Interestingly, the current study shows that 25(OH)D_3_ concentration 15 min and 1 h after the exercise increased by 4% overall, not considering the type of exercise or the biological age of the boys (p = 0.016, p = 0.011 respectively). It is important to note that all the boys were vitamin D sufficient. Therefore this seems to be a physiological response to physical activity. Several recent studies share similar observations where immediately after acute endurance exercises, the blood 25(OH)D_3_ concentration increased ^41,42^.

Interestingly, gender disparity was observed in the serum 25(OH)D_3_ response to acute endurance exercise and the increase in 25(OH)D_3_ concentrations immediately after the exercise was greater in men who had higher FFM than in women with higher fat percentage ^42^. In the present study, the increase of 25(OH)D_3_ concentration 15 min after the exercise was positively correlated to FFM with no connection to fat level. However, the alteration of the vitamin D concentration one hour after the exercise showed the exact opposite, the positive correlation of fat content and no connection to FFM. This might suggest that during the exercise, the muscular supplies of vitamin D are activated, whereas during the excess post-exercise oxygen consumption (EPOC), the released vitamin D comes from fat storage.

PTH is inversely related to 25(OH)D_3_, which insufficiency causes an increase in serum PTH ^43–45^. Hollick et al. ^44^ reported an inverse correlation between serum PTH and 25(OH)D_3_ concentrations in postmenopausal women. Another study has shown that in subjects supplemented with vitamin D serum, PTH concentrations dropped by ~ 20% ^46^. Nevertheless, PTH among with low Ca^2+^, and low PO_4_^3−^ concentrations activate 25-hydroxyvitamin D-1-α-hydroxylase (CYP27B1) ^47,48^, which functions to increase 1α,25(OH)_2_D_3_ synthesis from 25(OH)D_3_^11^. Therefore, a negative relationship exists between serum 25(OH)D_3_ and PTH. The threshold of serum 25(OH)D_3_, where serum PTH starts to rise, is about 75 nmol L^−1^, according to most studies ^49^. In our study, the group which reached the concentration of 78.8 nmol L^−1^ of 25(OH)D_3_ had the most noticeable increase of PTH concentration, which supports that phenomenon. Another study on young adolescent boys demonstrated that during pubertal growth and bone formation, low concentrations of 25(OH)D_3_ are associated with increased PTH and subsequently increased serum 1,25(OH)_2_D_3_. The purpose of these changes is to increase calcium absorption necessary for rapid bone-forming activity during adolescence ^50^. Moreover, a study on mice reports that the changes in whole bone mechanics during exercise are dependent on PTH signaling ^51^.

In the present study, the increase in 25(OH)D_3_ concentration was the most significant 15 min after WAnT in pubertal boys, and it reached 6.5% (p = 0.031). Interestingly, it was associated with a significant increase in PTH concentration in this group also 15 min after WAnT (p = 0.03). After the WAnT in younger boys, the increase was postponed, 15 min after the exercise, 25(OH)D_3_ concentration was similar to the level before, but after 1 h, the concentration of 25(OH)D_3_ increased by 4.6%. Also, PTH concentration in this group rose not 15 min after the test but 1 h after the exercise (Fig. 4). This finding may suggest that high-intensity interval exercise triggers the release of both 25(OH)D_3_ and PTH simultaneously. Since PTH activates CYP27B1, thus increasing the synthesis of 1α,25(OH)_2_ D_3_ from 25(OH)D_3_, it is possible that the part of release 25(OH)D_3_ was immediately transformed to 1α,25(OH)_2_D_3_. Active vitamin D may increase absorption of intestinal calcium and, therefore, contributes to the rise of calcium level, which finally reduces PTH. Future studies should address the whole spectrum of vitamin D metabolites to establish a flow of particular substances.

The increase in 25(OH)D_3_ concentration after the VO_2max_ test was similar in pre-pubertal and pubertal boys and reached ∼ 4.5%. This increase was not associated with PTH changes. The lack of linkage in PTH and vitamin D release after the VO_2max_ test might be due to a different exercise intensity. This result supports the data showing no effect of BMD of low-impact endurance exercise. We have observed several major differences between the two bouts. While lactate concentration after the VO_2max_ test averaged 3.4 ± 0.9 mmol L^−1^, after the WAnT, it averaged 9.9 ± 1.3 mmol L^−1^ after the first round, 10.5 ± 1.2 mmol L^−1^ after the second round, and 7.6 ± 1.2 mmol L^−1^ 1 min after the third round. Fifteen minutes after the VO_2_max test, lactate was already at the baseline level, while after WAnT it was maintained at the 8.6 ± 2.5 mmol L^−1^ concentration (Table 1). Moreover, our study shows a different response in IL-6 concentration after the tests. We have observed a rise of IL-6 in all groups 15 min after the tests. However, after WAnT, the peak increase of IL-6 occurred one hour after the exercise, while one hour after the VO_2_max test, IL-6 was already at the baseline level. What is most important the highest IL-6 concentration was found in the pubertal group, the same where we observed the highest output of 25(OH)D_3_. The delayed IL-6 peak after WAnT suggests a higher intensity of this test when compared to the VO_2max_ test. IL-6 is well documented to be stimulated by contracting muscle ^52–54^. Therefore Il-6 is not only a cytokine but also a myokine with an anti-inflammatory capacity ^54^. It was initially thought that the IL-6 response was related to muscle damage. However, it has become evident that muscle damage is not required to increase plasma IL-6 during exercise. Instead, eccentric exercise may result in a delayed peak and a slower decrease in plasma IL-6 during recovery ^54^. In contrast, the IL-6 response is sensitive to the exercise intensity ^55^, indirectly representing the muscle mass involved in the contractile activity. Another study on young males showed that single bout plyometric exercise and intermittent running acute exercises give different responses in serum bone formation markers, in favor of plyometric exercise, and suggest the changes might be due to an exercise-induced mechanical impact rather than bone cellular activities ^56^.

Furthermore, it has been shown that adipose tissue may both regulate and be regulated by vitamin D ^57^. Although vitamin D deficiency was shown to be associated with dyslipidemia, the short time effects of vitamin D on lipid metabolism are potentially unfavorable. A randomized controlled trial showed that vitamin D repletion cause a significant increase in LDL cholesterol ^58,59^, increased triglycerides, very-low-density lipoprotein-, low-density lipoprotein-, and high-density lipoprotein-triglycerides, as well as apolipoproteins B, CII, and CIII ^59^. Moreover, some experimental data suggest that vitamin D could promote greater adiposity, leading to elevated PTH, which may promote calcium influx into adipocytes, thereby enhancing lipogenesis ^60^. However, those trials include vitamin D treatment solely, with no exercise intervention. The present study showed the greatest boost of NEFA with a high glycerol extraction in the group with the most significant 25(OH)D_3_ release after WAnT. NEFA and glycerol are the products of lipolysis. Although during interval exercises, energy is delivered more from carbohydrates than lipids, in the EPOC, after interval exercise, elevated fat oxidation occurs ^61^. A recent study presented higher metabolic responses and a shift of substrate use toward lipids after the interval spinning exercise compared to isocaloric continuous exercise during the recovery period ^62^. As noted before, the difference between the WAnT and VO_2_max test may lay in the higher intensity and mechanical input of interval exercise. Therefore, it is not surprising that after the VO_2_max test, we did not find any significant rise neither in NEFA nor in glycerol. The rise in 25(OH)D_3_ concentration after the VO_2_max test was also less significant than after the WAnT. Therefore, it seems that the immediate post exercise release of vitamin D comes mainly from the working muscles, whereas fat storage is activated only during the recovery period.

Lastly, participants of the present study were boys, who trained soccer 2–3 times per week, who were asked to withdraw from the intensive exercise for 48 h before the tests. At the moment of the experiment, their organisms were in the training process. Nevertheless, both WAnT and VO_2max_ tests could change the 25(OH)D_3_ concentration. Previously mentioned studies ^41,42^ show that the increase of 25(OH)D_3_ concentration was maintained for 24 h after acute endurance exercises and that resistance ^9^ and endurance ^39^ training can enhance the change in the blood 25(OH)D_3_ concentration. It is well documented that acute training cannot cause as profound adaptive response in the organism as training does. However, considering that an increase after a single workout might cumulate after repetition gives a new perspective to the physiology of bone formation and the pleiotropic function of vitamin D. Additionally, regulation of vitamin D status through exercise might be clinically important before assaying vitamin D supplementation.

Limitation of the study: a potential limitation of this study was a small group of participants, and a lack of other groups undertaking types of exercise, particularly resistance exercise and exercise with very low intensity. We also miss the follow-up results 24 h after the exercise. Furthermore, we did not monitor the kids’ diets. Another limitation of the study was the examination of only serum 25(OH)D_3_ instead of the whole spectrum of vitamin D and only one marker of inflammation status. The positive insight might also bring assessing the bioavailable (free) fraction of vitamin D with the vitamin D binding protein assessment.

## Conclusion

We demonstrate the positive impact of acute exercise on increased 25(OH)D_3_ concentration in serum in young trained boys. The elevated 25(OH)D_3_ concentration in pubertal boys after the WAnT was associated with an increased level of PTH in the serum. Moreover, the assessments of response to the exercises found though the changes in the concentration of 25(OH)D_3_, PTH, NEFA, and glycerol were associated with the augmented concentration of IL-6 in both pubertal and pre-pubertal boys. Intensive interval exercise brings more potent stimuli to vitamin D metabolism in young organisms. Our results support the hypothesis that muscles may both store and release of 25(OH)D_3_. Moreover, it is possible that the regular physical activity, to which the participants were subjected before they took the tests, was a necessary factor allowing muscles to build a proper reservoir of 25(OH)D_3_.

Future studies are necessary to elucidate whether regular physical exercise implemented at a young age impacts vitamin D storage and regulation in skeletal muscle and the possible consequences for the protection against muscle atrophy in the aging-dependent process. Moreover, future studies should address the impact of exercise-induced vitamin D fluctuations on the bone formation process.

## Supplementary Information

Supplementary Information.
