# Supplementary material for: Single bout of exercise triggers the increase of vitamin D blood concentration in adolescent trained boys: a pilot study
Source: Sci Rep. 2022 Feb 3;12:1825. doi: 10.1038/s41598-022-05783-x (PMC8814171; doi:10.1038/s41598-022-05783-x)
Supplement: Supplementary file 1 — Supplementary Information. [file 41598_2022_5783_MOESM1_ESM.docx]

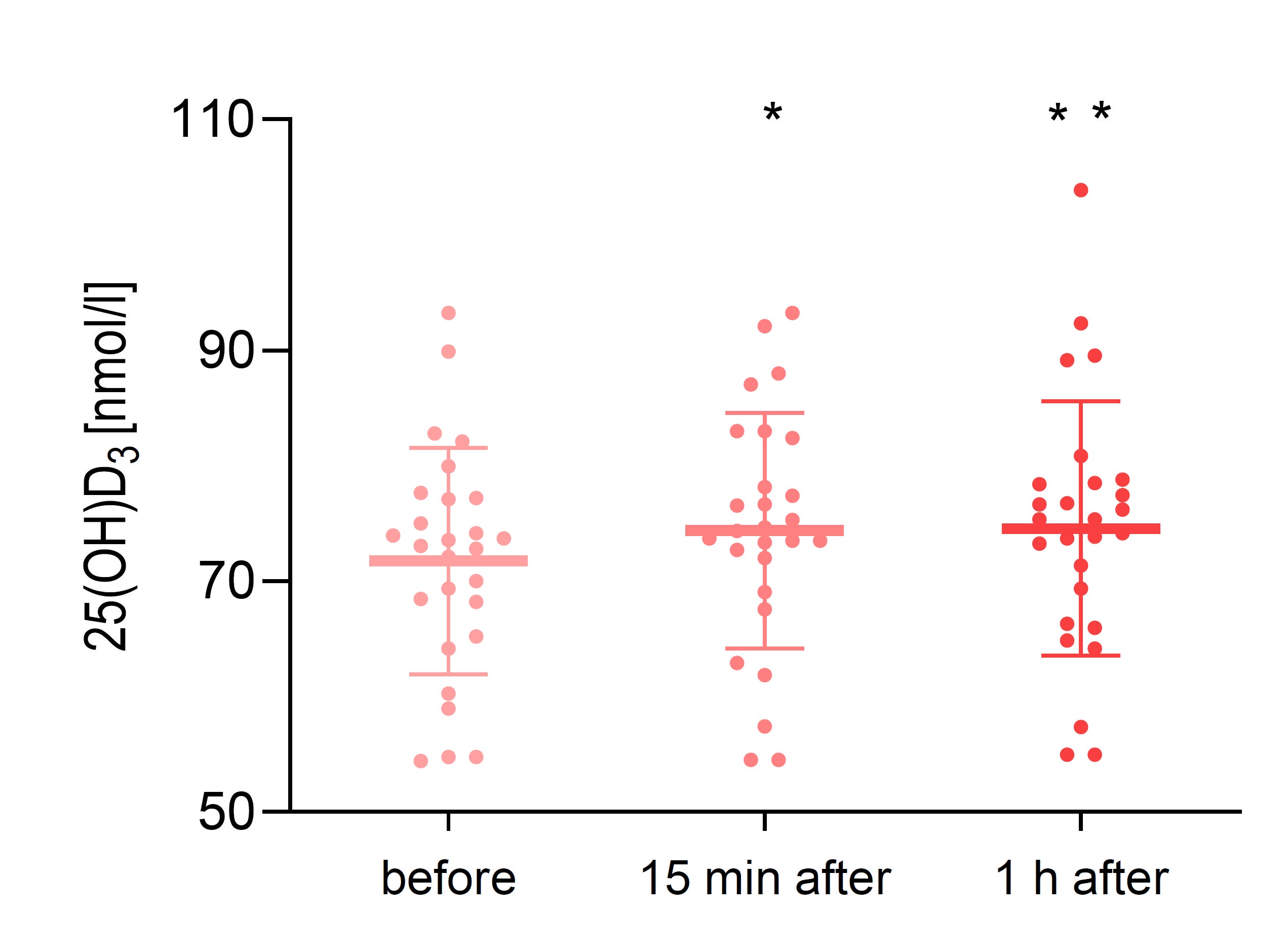


Figure S1. The concentration of 25(OH)D_3_ before, 15 minutes after and 1 hour after a single bout of exercise (both VO_2_max and WAnT tests) in boys n=26 (10-14 y). Results were expressed as mean±SEM. *p=0.016– difference between indicated result/mean and the results before, **p=0.011 – difference between indicated result/mean the results before, LSD *post hoc* test after repeated measures ANOVA.


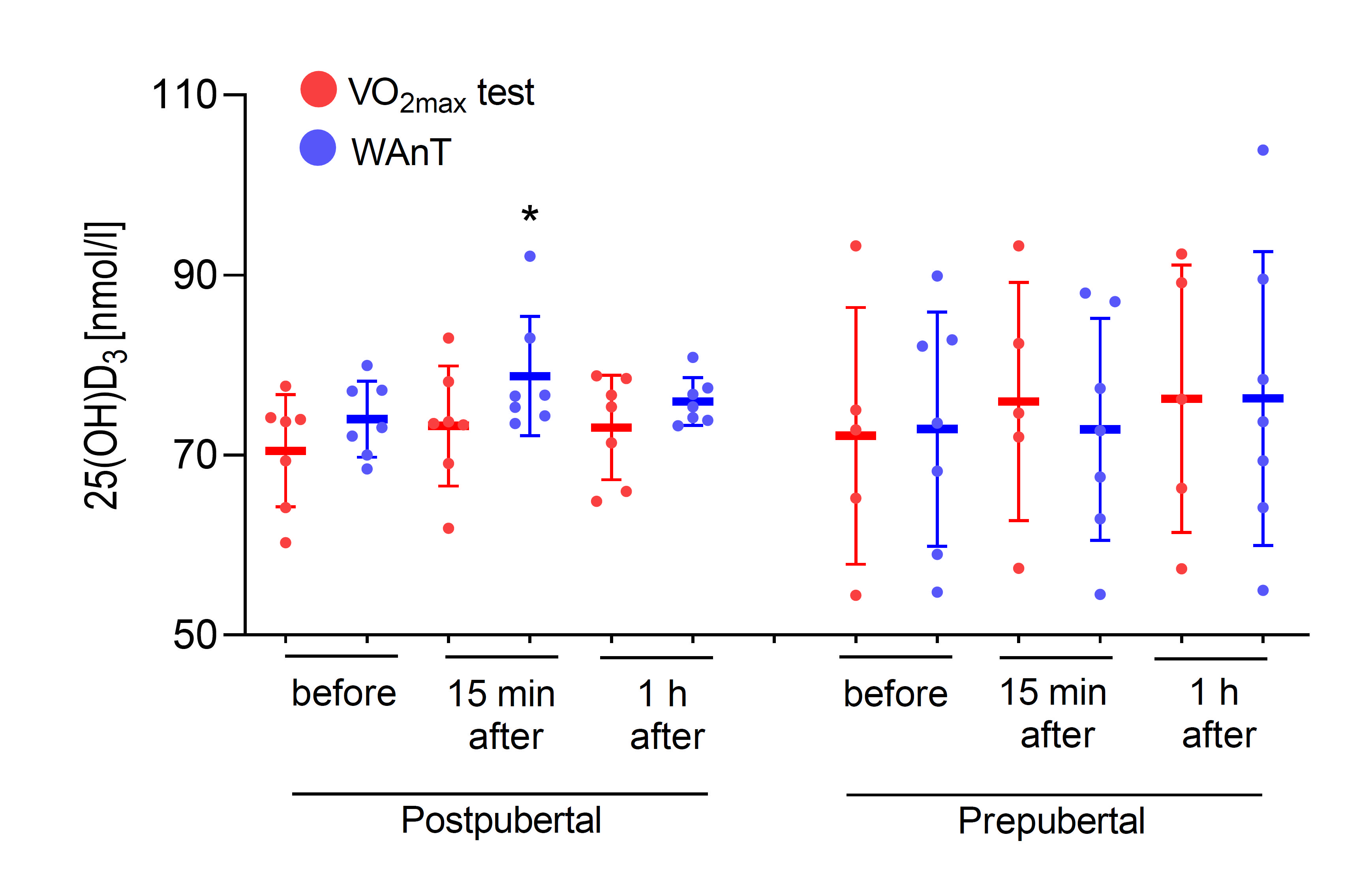


Figure S2. The concentration of 25(OH)D3 before, 15 minutes after and 1 hour after a single bout of exercise in pubertal (n= 7 for VO_2_max test, and n= 7 for WAnT test) and prepubertal (n= 5 for VO_2_max test, and n=7 for WAnT test) boys according to two types of exercise (VO_2_max and WAnT tests). *p=0.032 – difference between indicated result/mean and the results before in the same group and test, LSD *post hoc* test after repeated measures ANOVA.


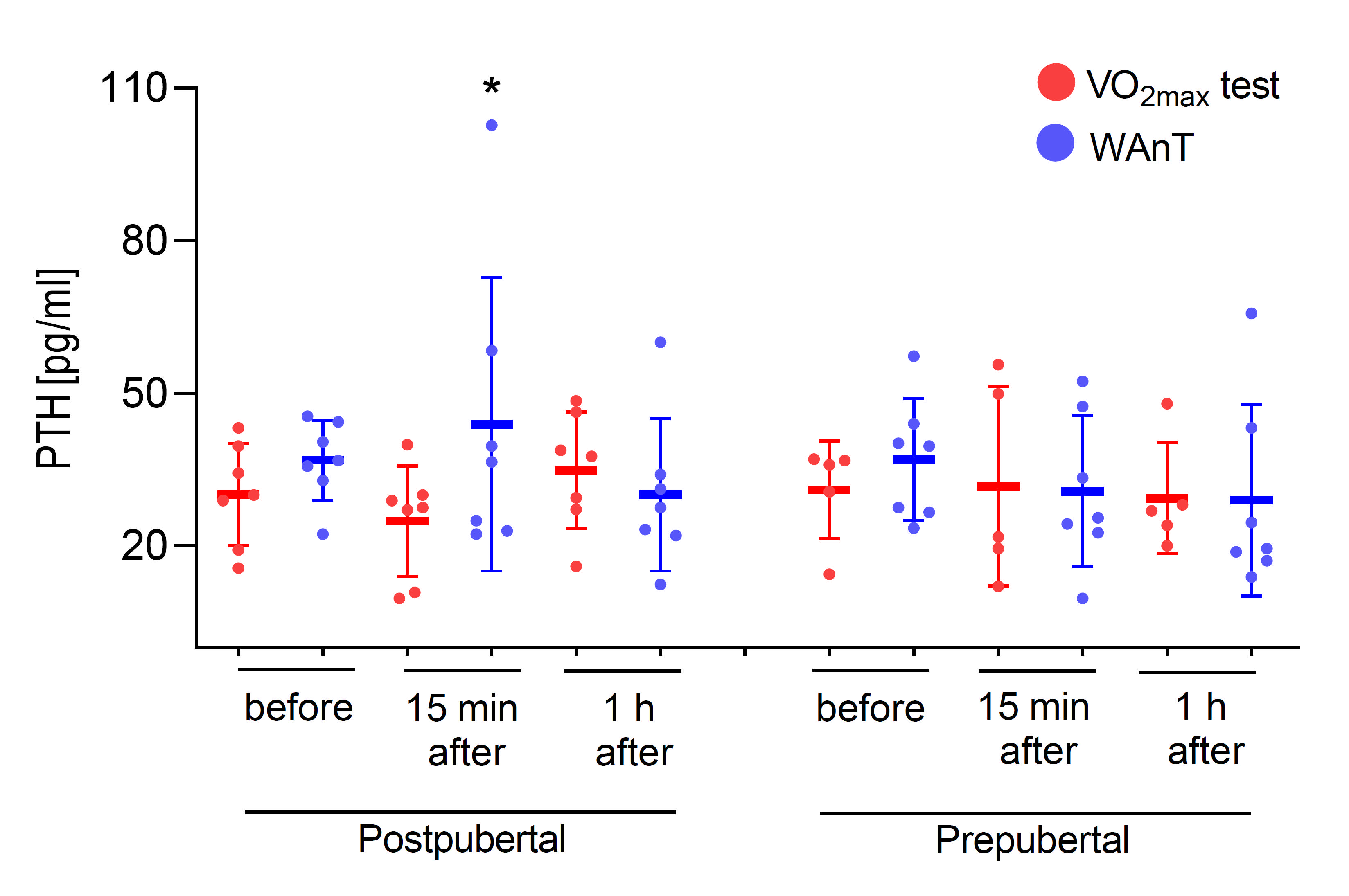


Figure S3. The PTH concentration before, 15 minutes after and 1 hour after a single bout of exercise in pubertal (n= 7 for VO_2_max test, and n= 7 for WAnT test) and prepubertal (n= 5 for VO_2_max test, and n=7 for WAnT test) boys according to two types of exercise (VO_2_max and WAnT tests). *p=0.024 – difference between indicated result/mean and the results 1 hour after in the same group and test, LSD *post hoc* test after repeated measures ANOVA.
